# Developing a national dissemination plan for collaborative care for depression: QUERI Series

**DOI:** 10.1186/1748-5908-3-59

**Published:** 2008-12-31

**Authors:** Jeffrey L Smith, John W Williams, Richard R Owen, Lisa V Rubenstein, Edmund Chaney

**Affiliations:** 1 VA Mental Health Quality Enhancement Research Initiative, Central Arkansas Veterans Healthcare System, 2200 Fort Roots Drive, Building 58 (152/NLR), North Little Rock, Arkansas, 72114, USA; 2 Durham VA Medical Center HSR&D, 508 Fulton Street (Building 16), Durham, North Carolina, 27705, USA; 3 VA HSR&D Center for the Study of Healthcare Provider Behavior, VA Greater Los Angeles Healthcare System, 16111 Plummer Street (Building 25), Sepulveda, California, 91343, USA; 4 VA HSR&D Center of Excellence, VA Puget Sound Healthcare System, 1100 Olive Way, Suite 1400, Seattle, Washington 98101, USA

## Abstract

**Background:**

Little is known about effective strategies for disseminating and implementing complex clinical innovations across large healthcare systems. This paper describes processes undertaken and tools developed by the U.S. Department of Veterans Affairs (VA) Mental Health Quality Enhancement Research Initiative (MH-QUERI) to guide its efforts to partner with clinical leaders to prepare for national dissemination and implementation of collaborative care for depression.

**Methods:**

An evidence-based quality improvement (EBQI) process was used to develop an initial set of goals to prepare the VA for national dissemination and implementation of collaborative care. The resulting product of the EBQI process is referred to herein as a "*National Dissemination Plan*" (NDP). EBQI participants included: a) researchers with expertise on the collaborative care model for depression, clinical quality improvement, and implementation science, and b) VA clinical and administrative leaders with experience and expertise on how to adapt research evidence to organizational needs, resources and capacity. Based on EBQI participant feedback, drafts of the NDP were revised and refined over multiple iterations before a final version was approved by MH-QUERI leadership. 'Action Teams' were created to address each goal. A formative evaluation framework and related tools were developed to document processes, monitor progress, and identify and act upon barriers and facilitators in addressing NDP goals.

**Results:**

The National Dissemination Plan suggests that effectively disseminating collaborative care for depression in the VA will likely require attention to: Guidelines and Quality Indicators (4 goals), Training in Clinical Processes and Evidence-based Quality Improvement (6 goals), Marketing (7 goals), and Informatics Support (1 goal). Action Teams are using the NDP as a blueprint for developing infrastructure to support system-wide adoption and sustained implementation of collaborative care for depression. To date, accomplishments include but are not limited to: conduct of a systematic review of the literature to update VA depression treatment guidelines to include the latest evidence on collaborative care for depression; training for clinical staff on TIDES (Translating Initiatives for Depression into Effective Solutions project) care; spread of TIDES care to new VA facilities; and integration of TIDES depression assessment tools into a planned update of software used in delivery of VA mental health services. Thus far, common barriers encountered by Action Teams in addressing NDP goals include: a) limited time to address goals due to competing tasks/priorities, b) frequent turnover of key organizational leaders/stakeholders, c) limited skills and training among team members for addressing NDP goals, and d) difficulty coordinating activities across Action Teams on related goals.

**Conclusion:**

MH-QUERI has partnered with VA organizational leaders to develop a focused yet flexible plan to address key factors to prepare for national dissemination and implementation of collaborative care for depression. Early indications suggest that the plan is laying an important foundation that will enhance the likelihood of successful implementation and spread across the VA healthcare system.

## Background

Little is known about effective strategies for disseminating and implementing complex clinical innovations across large, healthcare systems [1]. In their extensive review of the literature on diffusing innovations in healthcare organizations, Greenhalgh and colleagues acknowledged the dearth of research and empirical findings in this area for healthcare organizations, concluding that one of the most "striking findings" of their review was the "tiny proportion of empirical studies that acknowledged, let alone explicitly set out to study, the complexities of spreading and sustaining innovation in [health] service organizations" [2]. Strategies to facilitate spread or 'scale-up' of effective programs have been studied more extensively in other sectors, including public health [3,4], education [5], and child and family services [6].

Based on results from an effort to spread an evidence-based HIV prevention program across multiple communities, Rebchook and colleagues suggested that mechanisms be created for program developers to help agencies (implementers) modify or 're-invent' evidence-based programs appropriately so the program can be implemented with fidelity [3]. Additional factors identified for facilitating successful spread of evidence-based programs have included leadership support [5,7], staff training [4,5,7], and the development or optimized use of organizational infrastructure to support program implementation [5,7]. Though the direct applicability of lessons from other healthcare sectors to traditional care delivery systems such as the VA requires further study, there is at least face validity that such factors may be important for facilitating successful spread of evidence-based programs in such systems. Indeed, relevant conceptual models have identified similar factors as important considerations in implementing and sustaining evidence-based programs in healthcare organizations [2,8].

Innovative partnerships between researchers and organizational leaders represent a promising approach for addressing factors that may influence the successful spread of evidence-based programs in healthcare organizations [1,7,9-13]. Ross and colleagues describe three models of organizational decision-maker involvement in implementation research: 1) Formal supporter – provides explicit support for research goals but is not informed about or actively involved in the research process; 2) Responsive audience – responsive to researcher efforts to inform or engage them in the research process; or 3) Integral partner – engaged as a significant partner in the research process, primarily but not exclusively involving decision-maker initiated activities [9]. In Canada, health researchers report a broad range of dissemination and implementation activities in partnering with stakeholders in knowledge translation (KT) research [12]. Dissemination activities include the preparation of evidence summaries for policymakers [10,11], practitioners and patients, in addition to press releases, newsletters, and targeted mailings [12]. Implementation activities include educational sessions with practitioners, policymakers and patients; involvement of stakeholders and media in KT research; creation of tools; and the use of knowledge brokers [12]. In the United States, the Agency for Healthcare Research & Quality (AHRQ) has established practice-based research networks and other mechanisms to support partnerships between researchers and practitioners in order to enhance the uptake of evidence-based practices [13,14]. To fulfill the promise of research-clinical partnerships in enhancing the uptake and spread of evidence-based practices, it is important to identify tools and processes that can help support and optimize those partnerships [1]. According to a recent assessment of AHRQ activities in this area, successful partnerships between researchers and healthcare systems to encourage the uptake of research evidence require clear goals and appropriate targeting of resources [14].

This article is one in a *Series *of articles documenting implementation science frameworks and tools developed by the U.S. Department of Veterans Affairs (VA) Quality Enhancement Research Initiative (QUERI). QUERI is briefly outlined in Table 1 and described in more detail in previous publications [15,16]. The *Series' *introductory article [17] highlights aspects of QUERI related specifically to implementation science and describes additional types of articles contained in the *QUERI Series*.

**Table 1:** The VA Quality Enhancement Research Initiative (QUERI)

The U.S. Department of Veterans Affairs' (VA) Quality Enhancement Research Initiative (QUERI) was launched in 1998. QUERI was designed to harness VA's health services research expertise and resources in an ongoing system-wide effort to improve the performance of the VA healthcare system and, thus, quality of care for veterans.

QUERI researchers collaborate with VA policy and practice leaders, clinicians, and operations staff to implement appropriate evidence-based practices into routine clinical care. They work within distinct disease- or condition-specific QUERI Centers and utilize a standard six-step process:
1) Identify high-risk/high-volume diseases or problems.
2) Identify best practices.
3) Define existing practice patterns and outcomes across the VA and current variation from best practices.
4) Identify and implement interventions to promote best practices.
5) Document that best practices improve outcomes.
6) Document that outcomes are associated with improved health-related quality of life.

Within Step 4, QUERI implementation efforts generally follow a sequence of four phases to enable the refinement and spread of effective and sustainable implementation programs across multiple VA medical centers and clinics. The phases include:
1) Single-site pilot,
2) Small-scale, multi-site implementation trial,
3) Large-scale, multi-region implementation trial, and
4) System-wide rollout.

Researchers employ additional QUERI frameworks and tools, as highlighted in this *Series*, to enhance achievement of each project's quality improvement and implementation science goals.

This paper describes processes undertaken and tools developed by VA's Mental Health Quality Enhancement Research Initiative (MH-QUERI) to develop a plan to disseminate and spread collaborative care for depression (an evidence-based primary care depression treatment model [18-20]) across the VA nationally, in collaboration with clinical operations leaders and other key stakeholders. Specifically, the paper describes MH-QUERI's development of a National Dissemination Plan (NDP) to address organizational, policy and structural factors that might influence the uptake and sustainability of collaborative care for depression. In regard to activities described in this paper, organizational and clinical leaders are viewed as *integral partners *(see Ross, et al. definition above [9]) with researchers in the dissemination and implementation process. The paper also presents a framework and related tools for formative evaluation that was developed to document, optimize and evaluate activities undertaken to address National Dissemination Plan goals. Preliminary results from the formative evaluation on initial progress in achieving National Dissemination Plan goals and barriers encountered also are provided.

### Collaborative Care for Depression and the QUERI Six-Step Process

This section uses the QUERI Six-Step Process (see Table 1) [17] as a framework to briefly summarize: the prevalence and costs of depression within the VA healthcare system (Step 1), evidence for collaborative care as a 'best practice' for enhancing primary care depression treatment (Step 2), and gaps in quality of care for depression in the VA healthcare system (Step 3) that may be addressed through implementation of evidence-based care models. This section also summarizes MH-QUERI research to date on implementing collaborative care for depression as an evidence-based approach to enhance depression treatment in the VA healthcare system (QUERI Process, Steps 4,5+6).

### QUERI Step 1: Identify high-risk/high-volume diseases or problems

Approximately 7% of VA patients have a depression diagnosis [21,22], and patients with depression account for 14.3% of total VA healthcare costs [21]. Thus, depression is highly prevalent within VA treatment settings, imparting significant morbidity to patients and burden to the system in terms of resource expenditure and costs.

### QUERI Step 2: Identify best practices

Depression treatment guidelines jointly developed by the Veterans Health Administration and the U.S. Department of Defense recognize the clinical- and cost-effectiveness of collaborative care for depression [23], which has been shown in multiple efficacy and effectiveness trials to significantly improve depression treatment and outcomes for primary care patients with depression [18-20]. Table 2 outlines the key features and components of the collaborative care model. Although modest variation exists in features of collaborative care models shown to be effective in improving primary care depression treatment, the features listed in Table 2 have been recognized as common elements in models with demonstrated effectiveness [18-20].

**Table 2:** Key features of collaborative care for depression

Collaborative care for depression is an integrated package of tools and strategies that typically includes:

▪Clinician education and decision support for primary care providers
▪Depression Care Managers (typically primary care nurses) trained to:
◦ provide patient education, support patient self-management, identify treatment preferences, monitor adherence and side effects, and assess patient outcomes;
◦ communicate information on treatment adherence and outcomes to primary care and mental health clinicians; and
◦ facilitate communication among patients, primary care providers and mental health clinicians.

### QUERI Step 3: Define existing practice patterns and outcomes across the VA and current variation from best practices

Of VA patients screening positive for depression, only about half (54%) receive the recommended follow-up evaluation to confirm the diagnosis [24]. Another recent study found that among VA patients with severe depression symptoms, 36% remained undiagnosed and untreated with antidepressants over one year [25]. Further, veterans receiving depression treatment solely in VA primary care are less likely to receive antidepressants than veterans receiving some or most of their depression treatment in mental health specialty settings [26]. In addition, only 54% of VA patients started on antidepressants take the medication for the guideline-recommended duration of six months [27]. Failure to take antidepressants for the recommended duration has been shown to be a significant predictor of subsequent hospitalisation [28]. Although the quality of depression treatment in the VA is as good or better than that provided in other healthcare settings [29], there remains room for improvement.

### QUERI Steps 4/5/6: Identify and implement interventions to promote best practices; document that best practices improve outcomes; document that outcomes are associated with improved health-related quality of life

In health care, disruptive technologies are those innovations that may have major effects on important care processes, enable increased adoption of evidence-based care, or offer substantial improvements by disrupting or displacing previous systems of care [30]. Collaborative care for depression can be characterized as a complex clinical innovation and 'a disruptive technology' [30] because it represents a significant departure from current approaches for treating depression in primary care, such as new roles for clinical staff, systematic monitoring of treatment adherence and outcomes, and enhanced collaboration between primary care and mental health clinicians.

The above suggests a potentially higher level of difficulty in achieving the successful implementation of collaborative care for depression across a large healthcare system because it requires considerable system resources and significant demands for change at the local level. The VA may be particularly well suited for the challenge of implementing disruptive technologies through its organizational capacity to provide clinical practice guidelines, performance measurement, staff training, and computerized decision support. In addition, implementation expertise and facilitation through a QUERI Center is available to help leverage and enhance system resources that support implementation and spread [17,31].

An initial MH-QUERI research project titled Translating Initiatives for Depression into Evidence-Based Solutions (TIDES) implemented collaborative care for depression in six primary care clinics located across three VA healthcare networks. In reference to the 4-phase implementation research framework described in the Overview article [17], TIDES was a Phase 2 demonstration project involving a modest number of VA networks and facilities for adaptation, refinement, and ongoing support of collaborative care for depression. In this case, Phase 1 testing (small-scale pilot; see Table 1) of collaborative care was skipped because those studies are typically undertaken to assess potential barriers or needed toolkits for newly developed interventions – or to adapt interventions developed outside of VA for initial feasibility testing in VA treatment settings. Because an earlier effectiveness trial (Step C) had already demonstrated the initial feasibility and effectiveness of collaborative care in VA treatment settings [8,16], there was no need for Phase 1 testing.

TIDES used an evidence-based quality improvement (EBQI) process [32] to facilitate collaboration among researchers, network leaders, and clinicians to customize implementation of collaborative care for depression in diverse VA treatment settings. The EBQI process allows tailoring of collaborative care implementation to local priorities and resources, while maintaining fidelity to the evidence base for model design [32]. The TIDES collaborative care model for depression included telephone assessment and follow-up by a Depression Care Manager (DCM), treatment plans based on depression algorithms selected by the primary care physician (PCP), supervision of the DCM by a mental health specialist (MHS), and consultation between the MHS and PCP as needed. Evaluation results have shown implementation of the Phase 2 TIDES collaborative care process to be successful. Among patients referred to depression care managers, 82% were treated for depression in primary care and 74% stayed on medication; and 90% of primary care patients and 50% of mental health patients had clinically significant reductions in depressive symptomatology at six-month follow-up [33-36].

In follow-up to the Phase 2 TIDES success, a larger-scale Phase 3 demonstration project [17] referred to as Regional Expansion of TIDES (ReTIDES) was undertaken to: a) sustain TIDES collaborative care at VA sites implementing the model in the Phase 2 study described above, b) spread TIDES collaborative care regionally to new VA networks and clinics, and c) prepare the VA healthcare system for a Phase 4 "national roll-out" effort [17]. As noted earlier, this paper focuses specifically on the last objective, such as MH-QUERI's pro-active efforts to partner with organizational leaders to develop a National Dissemination Plan during the Phase 3 demonstration project. The purpose of the Plan is to prepare the VA healthcare system for national dissemination and implementation of collaborative care, wherein the delivery model for depression would be taken forward by VA clinical operations in the Phase 4 roll-out [17]. During Phase 4, the MH-QUERI team would collaborate with VA clinical leaders to facilitate the spread and sustainability of collaborative care, if indicated per Phase 3 evaluations.

Terminology and nomenclature are important in VA's QUERI program, which has adopted standard definitions for commonly used terms in our implementation research to facilitate communication and enhance learning across groups (see Table 1 in Stetler et al [17]). Consequently, it is important to point out that although the Phase 3 project team chose the shorter title of "National Dissemination Plan," its collection of goals clearly represent both dissemination and implementation activities on the part of researchers to help spread collaborative care for depression across the VA.

## Methods

### Organizing the Project

The MH-QUERI Executive Committee – comprised of research experts and VA organizational leaders in the areas of mental health, clinical quality improvement and organizational change – serves an oversight function and plays an active role in guiding strategic planning and implementation research pertaining to the MH-QUERI mission [15,17]. At the outset of the Phase 3 ReTIDES project mentioned above, the Executive Committee established a subcommittee referred to as the Depression Subgroup (DSG) and charged it with developing a plan to prepare the VA for national dissemination of collaborative care for depression. The DSG is comprised of MH-QUERI Executive Committee members and other individuals who were selected for membership based on their expertise in implementation research, depression treatment, implementation of collaborative care for depression, and/or because they were key VA clinical leaders or stakeholders in the areas of mental health service delivery or quality improvement.

To provide guidance to the DSG on issues pertaining to sustainability, a ReTIDES Steering Committee was established as a stipulation of the VA funding agency to oversee project planning and implementation. The Steering Committee's function is to serve an advisory and problem-solving role to the DSG and ReTIDES investigative teams, while also exercising overall project governance (e.g., monitoring progress in achieving project objectives). Its membership is comprised of individuals with implementation research expertise and/or with VA organizational experience and implementation insights. Various Steering Committee members also were in a position to facilitate networking and linkage with potential partners who might be helpful in preparing the VA system for national roll-out.

### Developing the National Dissemination Plan

Development of the TIDES National Dissemination Plan (NDP) followed an adapted evidence-based quality improvement (EBQI) process [32]. EBQI takes advantage of features known to facilitate innovation, including directly working through decision-making processes with organizational stakeholders, allowance for contextual adaptation that does not result in deviations from the evidence base, and involvement of researchers as change agents [1,32,37-39]. The goal of EBQI in this effort was to foster a partnership between VA researchers and organizational leaders to develop a plan that would identify goals for addressing important policy and structural factors that may impact national dissemination of collaborative care.

EBQI participants included: a) researchers with expertise on the collaborative care model for depression, clinical quality improvement, and implementation science; and b) VA clinical and administrative leaders with experience and expertise on how to adapt research evidence to organizational needs, resources, and capacity. EBQI participants on the research side of the partnership included DSG members who either volunteered or were asked by MH-QUERI leadership to participate in the process. EBQI participants on the clinical/administrative side included VA leaders in mental health service delivery, guideline development, performance measurement, and nursing. Clinical/administrative EBQI participants were identified purposively (based on their position in the VA organizational structure) or through recommendations from other EBQI participants, or the ReTIDES Steering Committee. Operationalization of the EBQI process for developing the national dissemination plan involved the following five steps.

1. DSG leaders prepared an initial draft of NDP goals, drawing upon their expertise in implementing collaborative care and knowledge of structural supports likely to be necessary to support national dissemination [40-42]. In regard to the latter, a recent large-scale initiative to disseminate evidence-based primary care depression treatment models found that the most important factors impacting program sustainability were: more specific clinical practice guidelines, meaningful quality indicators, funding and structural mechanisms to support technical assistance, staff training, and clinical information system enhancements [40,41]. Similarly, in a recent randomized controlled trial to implement and test collaborative care for depression in older adults, investigators identified four key determinants of program sustainability (defined as continuation of all or part of the collaborative care model as 'usual care' up to one year after the research project concluded): 1) demonstration of positive clinical outcomes, 2) institutional support (strong leadership support to continue the program), 3) trained staff, and 4) continued funding sources to support implementation [42]. The ability to adapt implementation of collaborative care to enhance fit with the context of the organization also was associated with program sustainability [42].

It is important to note that a specific conceptual framework was not selected to explicitly guide this EBQI process or development of the NDP. Instead, EBQI participants drew upon their implementation research, clinical and/or organizational expertise, as well as knowledge of findings from related large-scale initiatives [40-42] in drafting NDP goals. Goals in the initial NDP draft also reflected feedback from select VA leaders regarding important factors to address in national dissemination planning.

2. The initial draft of the NDP was distributed to the broader group of DSG members as well as the ReTIDES Steering Committee for review and feedback, with both groups including implementation research experts and organizational leaders at the national level.

3. A revised draft of the NDP was distributed to EBQI participants via e-mail for their review and comments/suggestions for further refinement.

4. Using a modified Delphi process [43], the NDP was then refined based on feedback from EBQI participants over multiple iterations. Comments and suggestions from EBQI participants were compiled and incorporated into revised versions over approximately a three-month period, until consensus was reached on a final draft.

5. The final draft of the NDP from EBQI participants was reviewed and approved by the MH-QUERI Executive Committee.

### Forming action teams to address national dissemination plan goals

DSG leaders reviewed the National Dissemination Plan goals and identified ten individuals with suitable expertise to lead 'Action Teams' for each of the 18 NDP goals. [Note that the number of Action Team leaders (10) is less than the number of NDP goals (18) because some individuals served as Action Team leader for multiple goals.] Action Team leaders included DSG members and doctoral-level researchers involved with the ReTIDES project. Action Team leaders were instructed to assemble a team to assist in achieving their respective goals and to develop an Action Plan to address the goal.

Consistent with Complexity Theory [44-46], flexibility was built into the process through the use of general, non-prescriptive language to allow Action Teams to chart a course to achieve their goals. Additionally, the Action Teams were given the freedom to modify or refine their NDP goals as needed during the course of executing the plan – following review and approval by the DSG – based on subsequent experience, evolving circumstances in the VA, and/or feedback from ongoing formative evaluation (see below) [47]. Further information on guidance provided to NDP Action Team leaders on how to proceed in addressing NDP goals is provided in Table 3.

**Table 3:** Guidance for National Dissemination Plan Action Team Leaders

*Action Team assembly and composition*
▪ Team leaders were given discretion to assemble team members they believe could help them achieve the NDP goal, but were encouraged to consider including a DSG member and/or a ReTIDES investigator to help ensure coordination with related project activities.
▪ Team leaders were encouraged to consider the balance and value of involving researchers, clinicians, managers, VA leaders, technical/content experts, and/or consumer representatives on the Action Team.

*Developing an action plan*
▪ Team leaders were encouraged to draft an action plan and timeline for accomplishing the NDP goal. A draft action plan was provided to each team leader who was empowered to revise the plan as needed.
▪ Recognizing that action plans would need to be flexible and adaptable over time as new information emerged or organizational circumstances changed, team leaders were encouraged to not allow their team to be slowed by time intensive planning.

*Progress reporting and coordination across teams*
▪ Team leaders were informed that they would be asked to provide written, quarterly progress reports and to participate periodically in MH-QUERI conference calls.
▪ Team leaders were provided guidance on related NDP goals that may require coordination of efforts across different action teams.
▪ Team leaders were asked to be attentive to additional overlap issues that might emerge during the project that might require coordination.

### Formative evaluation framework and tools

Formative evaluation (FE) has been defined as a "rigorous assessment process designed to identify potential and actual influences on the progress and effectiveness of implementation efforts [47]." Formative evaluation data collection occurs before, during, and after implementation to optimize the potential for success by gaining a better understanding of the processes involved, identifying the need for refinements, and assessing the merit of using a similar approach in future dissemination and implementation efforts.

Recognizing the general value of formative evaluation (FE), the research team developed a goal-related FE framework and associated tools to monitor the DSG's creation and execution of the National Dissemination Plan. This was conducted concurrently and has helped to inform, refine and evaluate DSG activities and progress in achieving NDP goals. Table 4 lists our primary objectives for formative evaluation of the DSG. Additionally, the work of the MH-QUERI in preparing for a Phase 4 national roll-out was to serve as a precedent and template for other QUERI Centers as they progress along the implementation pipeline [17]. Thus, optimal information about the process from the FE with its related barriers and facilitators was critical.

**Table 4:** Formative evaluation objectives

▪ *Collect and feed back goal-related implementation and progress data to the Mental Health QUERI Depression Subgroup (DSG) on an ongoing basis *to concurrently evaluate, inform and identify needs for refinement of efforts to prepare the system for national dissemination and implementation of collaborative care.
▪ *Document the process of DSG activities *to create organizational infrastructure to support national dissemination.
▪ *Evaluate DSG success in meeting pre-specified goals and delivering pre-specified products *– see TIDES National Dissemination Plan (Table 5).
▪ *Evaluate DSG flexibility to successfully meet additional goals or create additional products that emerge as important during the process*, which were not conceived at the outset.
▪ *Identify key barriers and facilitators to the process *and document if/how they were overcome (barriers) or leveraged (facilitators).
▪ *Identify any unintended consequences *that emerge during the process.
▪ *Obtain perspectives of DSG members and key VA stakeholders on the success/value of research-clinical partnerships *to prepare the system for national dissemination of collaborative care.

Formative evaluation of DSG activities includes implementation-focused, progress-focused, and interpretive components [47]. The primary data source for implementation- and progress-focused FE are progress reports submitted by leaders of the Action Teams addressing NDP goals. A systematic process for quarterly progress reporting (i.e., report templates and distribution/reminder protocols) was developed to collect information on advancement toward achieving NDP goals. The Additional File shows the template developed to encourage consistency in quarterly progress reporting [Additional File 1]. Key information from these quarterly progress reports is condensed into a summary report to: a) allow the DSG and the Steering Committee to monitor progress in addressing NDP goals on a quarterly basis, b) identify barriers for problem-solving, c) ensure information exchange and coordination to minimize duplication of efforts and reduce the potential for unnecessarily burdening organizational stakeholders, and (d) help identify any modifications to the NDP that may be needed.

At the conclusion of ReTIDES, semi-structured qualitative interviews will be completed with all DSG members and select VA organizational stakeholders for an interpretive evaluation [47]. For the interpretive evaluation, we will collect data on: stakeholder experiences, perceptions of the success and value of the research-clinical partnership, satisfaction with the process, barriers and facilitators, and recommendations for similar efforts in the future. Although one of our primary objectives in this manuscript is simply to describe the *framework *and *tools *we have established for formative evaluation, we provide preliminary findings from the formative evaluation on progress achieved and barriers encountered in the Results section. However, it is important to note that the operationalization of each NDP goal and the formative evaluation is ongoing. Accordingly, final results from the formative evaluation will be presented in later publications.

## Results

### TIDES National Dissemination Plan and initial progress

A product of the EBQI process described above, the TIDES National Dissemination Plan (NDP; see Table 5) is a blueprint for developing infrastructure and organizational support for national dissemination and implementation of collaborative care for depression. The NDP includes 18 goals pertaining to four factors: 1) guidelines and quality indicators (4 goals), 2) training in clinical processes and evidence-based quality improvement (6 goals), 3) marketing (7 goals), and 4) informatics support (1 goal). Attention to factors such as these has been identified as important by other large-scale efforts to disseminate collaborative care models for depression [40-42], as well as implementation science literature [2,8]. Table 5 outlines the specific goals for each section of the NDP. As noted above, Table 5 represents the 'baseline' NDP, which is subject to revision in response to evolving circumstances over time (as needed). The goals for each section of the NDP are summarized below.

**Table 5:** TIDES National Dissemination Plan

The TIDES National Dissemination Plan (NDP) is a blueprint for developing infrastructure to support the system-wide adoption of collaborative care for depression. The NDP includes goals pertaining to: 1) guidelines and quality indicators, 2) training in clinical processes and evidence-based quality improvement, 3) marketing, and 4) informatics support.

**Guidelines and quality indicators**
**Goal 1: **Partner with relevant VA offices/entities to update clinical practice guidelines for depression to reflect the evidence base for collaborative care.
**Goal 2: **Encourage and support efforts for VA to adopt performance indicators that reward collaborative care for depression.
**Goal 3: **Create tools to assess fidelity to the TIDES collaborative care model.
**Goal 4: **Develop a process for MH-QUERI to serve as an ongoing advisor to relevant VA offices on depression performance indicators.

**Training in clinical processes and evidence-based quality improvement**
**Goal 1: **Develop materials and processes to train primary care clinicians, nurse care managers, and psychiatrists on TIDES collaborative care.
**Goal 2: **Explore feasibility of developing a certification process for depression care managers.
**Goal 3: **Identify and develop needed implementation tools and strategies.
**Goal 4: **Develop methods to identify depression opinion leaders within VA networks, and develop materials to train opinion leaders and clinical managers in evidence-based quality improvement processes.
**Goal 5: **Develop tools for assessing site needs prior to implementing TIDES collaborative care, assessing organizational readiness for change, and obtaining staff participation in tailoring interventions to site-specific needs.
**Goal 6: **Develop a process for partnering with the VA Employee Education System to make updates to TIDES educational materials, as needed.

**Marketing**
**Goal 1: **Develop a marketing plan to promote the spread of TIDES collaborative care to new VA networks and facilities.
**Goal 2: **Keep key VA leaders and stakeholders apprised of progress in spreading TIDES collaborative care to new VA networks and facilities.
**Goal 3: **Develop the business case for depression care management.
**Goal 4: **Disseminate scientific findings related to the implementation and evaluation of TIDES collaborative care through scientific meetings, newsletters, and peer-reviewed journals.
**Goal 5: **Recruit four new VA networks to begin implementing TIDES collaborative care by the end of 2007, and at least 15 more networks by 2010.
**Goal 6: **Secure funding to support MH-QUERI efforts to facilitate spread and sustainability of TIDES collaborative care.
**Goal 7: **Develop a cadre of experts in depression care management, evidence-based quality improvement, informatics, and logistics to serve as consultants on implementing TIDES collaborative care.

**Informatics support**
**Goal 1: **Develop capacity within the VA computerized patient record system for depression care managers to track patients and document care.

### Guidelines and quality indicators

This section of the NDP includes goals for updating VA depression treatment guidelines [23] (goal 1) and developing performance indicators that reflect current evidence for collaborative care as an effective model for primary care depression treatment [1] (goals 2 and 4). VA healthcare managers are accountable for achieving targets on selected, system-wide evidence-based performance indicators (through performance management plans and payment mechanisms), and such indicators can be a powerful motivator for implementation of evidence-based practices [48]. This section also includes a goal to create fidelity monitoring tools to ensure that collaborative care implementation remains faithful to the evidence base (goal 3).

Examples of initial progress on goals in this section include: 1) Action Team members assisted in organizing a panel to update VA clinical practice guidelines for depression to include the latest evidence for collaborative care for depression, involving preparation and subsequent publication of a systematic review of multifaceted interventions (including collaborative care models) to improve depression care [20] (goal 1); and 2) a program integrity tool was created to identify key features of collaborative care models for depression, and related performance targets for implementation (goals 2 and 3).

### Training in clinical processes and evidence-based quality improvement

This section lists goals to collaborate with VA organizational entities to develop training programs to educate managers and clinicians on the collaborative care model and depression care management processes (goals 1, 2 and 6). As noted above, collaborative care is a complex clinical innovation, involving new roles and responsibilities for primary care and mental health clinicians; thus, access to proven training programs and materials for clinical staff are vital to implementation [7]. This section of the NDP also includes goals to develop processes and tools to support tailoring of TIDES implementation to local resources, while maintaining fidelity to the critical features of program implementation (goals 3, 4 and 5) [3].

An example of initial progress on these goals is that Action Team members and ReTIDES investigators partnered with VA's Employee Education System (EES) to constitute an advisory board for depression care manager training, and have also hosted conferences to train depression care managers (goals 1 and 6). Further, Action Teams for goals 3, 4 and 5 have developed a collection of tools for clinical leaders' use in identifying local opinion leaders and obtaining staff feedback in tailoring TIDES implementation to local needs.

### Marketing

This section includes goals for developing marketing materials and strategies to: a) obtain leadership input and buy-in for the national dissemination strategy, b) promote the spread of TIDES collaborative care to new VA networks in Phase 4 implementation [17], c) keep VA leaders and advisory groups apprised of progress toward regional spread and national implementation, d) outline a business case for collaborative care to inform leadership decisions on resource allocation, and e) disseminate scientific findings related to TIDES implementation and evaluation. Goals to prepare a systematic review of collaborative care and to elicit input from VA leaders in developing marketing messages are consistent with approaches recommended by Lavis and colleagues to inform healthcare leaders' decision-making on the adoption of evidence-based practices [10,11]. These strategies also are consistent with social marketing approaches to engage targeted end-users in defining key messages to promote innovation adoption [49].

Examples of initial progress on Marketing goals include: 1) development and presentation of an assortment of informational tools and materials on TIDES collaborative care for dissemination to various stakeholder groups (e.g., brochures, fact sheets, briefing documents, Powerpoint presentations) (goals 1 and 2); and 2) as of February 2007, TIDES depression care management was being implemented at facilities in 10 VA healthcare networks (goal 5). The spread of TIDES was aided substantially by funding from the VA Office of Mental Health Services to implement evidence-based programs to integrate primary care and mental health services.

### Informatics support

This section lists a goal to develop informatics tools to leverage VA's computerized patient record system to support implementation of TIDES collaborative care. Specifically, informatics tools are needed to: a) support the establishment of depression registries, b) support depression care manager activities in monitoring patient outcomes and treatment adherence, c) facilitate evidence-based clinical decision-making, and d) enhance patient education and self-management. A recent systematic review concluded that computerized clinical decision support systems can improve practitioner performance on a range of clinical behaviors, including diagnosis, preventive care, disease management, and medication management [50]. Further, computerized clinical information system enhancements can be an important factor in sustaining evidence-based depression treatment models in primary care [40,41].

Examples of initial progress by the Action Team on this goal include: 1) development of informatics software that includes depression assessment screens, panel management features, and capacity to graph patient outcomes over time; and 2) consultation and support to mental health informatics developers to import depression assessment and structured follow-up data entry tools into a planned update of software used throughout the VA to support delivery of mental health services.

### Initial barriers to addressing national dissemination plan goals

In addition to documenting and evaluating initial progress on attainment of NDP goals, preliminary findings from the formative evaluation have identified the following barriers commonly experienced by Action Teams.

• Limited time for Action Team members to address NDP goals due to competing tasks and priorities, making it difficult to maintain contacts and nurture relationships with busy VA clinical leaders.

• Frequent turnover of key VA clinical leaders, requiring re-investment of researcher time and resources to engage with new leaders to secure support and assistance in addressing NDP goals.

• Limited skills and training among Action Team members for addressing NDP goals (e.g., Action Team members addressing NDP marketing goals have expressed concern about their lack of marketing skills and training).

• Difficulty coordinating activities across multiple Action Teams that are addressing related NDP goals and working with the same VA clinical leaders.

Information on common barriers experienced by the Action Teams – along with data on more 'goal-specific' barriers – is summarized in quarterly progress reports distributed to the MH-QUERI Depression Subgroup and the ReTIDES Steering Committee for discussion and problem-solving. The objectives of sharing this information with these groups are to keep them updated on progress toward attainment of NDP goals, generate recommendations on how to overcome barriers, and inform discussion and decision-making on whether there may be a need to refine or modify NDP goals. Initial progress on NDP goals indicates that the formative evaluation framework has been at least modestly successful in supporting Action Teams' efforts to address a range of NDP goals. Further, feedback from Action Team leaders has indicated that data collection and information-sharing from the formative evaluation have been helpful in facilitating communication and coordination across Action Teams (helping to address one of the barriers noted above).

## Conclusion

Implementation and spread of complex clinical innovations such as collaborative care for depression is not likely to occur through efforts to simply make organizational leaders and policymakers aware of their clinical effectiveness [40,41]. Partnerships between researchers and clinical leaders to attend to key policy and structural factors may help ensure that implementation is successful and sustainable over the long term [40-42]. Utilizing an evidence-based quality improvement process [32], MH-QUERI partnered with VA organizational leaders to develop a focused yet flexible plan to prepare the system for national dissemination and implementation of collaborative care. The National Dissemination Plan includes a total of 18 goals pertaining to four factors: 1) clinical practice guidelines and quality indicators, 2) training, 3) marketing, and 4) informatics support.

As noted above, a specific conceptual framework was not selected to explicitly guide the EBQI process or development of national dissemination plan goals. Instead, EBQI participants drew upon their own diversified implementation research and clinical expertise, as well as knowledge of relevant findings from similar large-scale dissemination initiatives [40-42]. This could be viewed as a potential limitation of the approach we have taken if EBQI participants have overlooked important constructs or factors explicated in one or more theoretical models on disseminating evidence-based practices in healthcare organizations. This issue and other potential limitations will be explored in formative and summative evaluation results to be reported in future publications.

Given our implementation research experiences to date [1,17,38,39,47,51], and in light of other research [9-12], we view the research-clinical partnership as vital to ensuring that necessary organizational infrastructure is in place to enable and support TIDES collaborative care – both in the short- and long-term. Researcher involvement in the partnership is critical to ensure that implementation is faithful to the evidence base and that an effective and feasible implementation strategy is offered to stakeholders [3]. Equally critical is the involvement of clinical leaders to ensure that implementation is in line with organizational priorities, is customizable to local conditions, and makes efficient use of resources. With its QUERI program, VA may be uniquely structured to support such research-clinical partnerships, although similar partnerships may be feasible in other healthcare systems with internal research programs or other systems or entities that are committed to establishing and supporting such relationships [1].

Although formative evaluation of efforts to address National Dissemination Plan goals is ongoing, results suggest that our approach has achieved some important successes and milestones to encourage uptake and spread of collaborative care for depression in the VA. NDP Action Team members assisted in organizing a panel and conducting a systematic review of the literature [20] to update VA clinical practice guidelines to include the latest evidence on collaborative care for depression. Action Team members also have partnered with VA employee education leaders to host trainings for clinical staff on TIDES care, influenced and supported spread of TIDES care to new VA facilities, and provided support for the integration of TIDES depression assessment tools into a planned update of software used in the delivery of VA mental health services.

The formative evaluation framework in this project is helping to monitor progress, identify barriers, and concurrently inform any needed refinements to the National Dissemination Plan. The formative evaluation serves multiple functions. By design, it serves a research function to document the processes undertaken to achieve National Dissemination Plan goals, identify barriers and facilitators, and ultimately, to evaluate success in achieving those goals (i.e., summative evaluation). In addition to the research function, the formative evaluation also is working to optimize Action Teams' efforts to address NDP goals through the systematic collection and timely feedback of data on progress achieved and barriers encountered. Interestingly, barriers encountered by Action Teams in their initial efforts to address NDP goals are similar to barriers reported by other researchers involved in knowledge translation activities [12], including lack of time due to competing priorities, lack of relevant skills/expertise to accomplish goals, and frequent turnover of key organizational partners. Data from the formative evaluation are used in support of problem-solving and/or refinements to the NDP, as needed. At the conclusion of the project, results from the formative evaluation may help advance our understanding of critical principles, concepts, and determinants to consider and address in planning for similar large-scale dissemination and implementation efforts.

Summative evaluation of the National Dissemination Plan, including its developmental processes and end products cannot occur until well into Phase 4 [17]. Upon its completion, important insights will be gained about national roll-out initiatives, including barriers and facilitators to implementation and sustainability. If the NDP proves successful as a tool to support the national dissemination and implementation of collaborative care for depression, it may be a useful strategy for other implementation researchers involved in large-scale initiatives to implement and spread complex clinical innovations.

## Competing interests

The authors declare that they have no competing interests.

## Authors' contributions

JLS conceived of the study, participated in its design and coordination, and drafted the manuscript. JWW helped conceive of the study, participated in its design and coordination, and provided consultation. RRO helped conceive of the study, participated in its design and coordination, and provided consultation. LVR helped conceive of the study, participated in its design, and provided consultation. EC helped conceive of the study, participated in its design, and provided consultation.

## Disclaimer

The views expressed in this article are those of the authors, who are responsible for its contents, and do not necessarily represent the views of the U.S. Department of Veterans Affairs.

## Supplementary Material

Additional file 1 **National Dissemination Plan Progress Reporting Form**. The file provides an example of the progress report template which served as a formative evaluation tool to encourage consistency in reporting on process and progress toward attainment of NDP goals, and barriers/facilitators encountered. Click here for file
