# Supplementary material for: Developing a national dissemination plan for collaborative care for depression: QUERI Series
Source: Implement Sci. 2008 Dec 31;3:59. doi: 10.1186/1748-5908-3-59 (PMC2631596; doi:10.1186/1748-5908-3-59)
Supplement: Additional file 1 — National Dissemination Plan Progress Reporting Form. The file provides an example of the progress report template which served as a formative evaluation tool to encourage consistency in reporting on process and progress toward attainment of NDP goals, and barriers/facilitators encountered. [file 1748-5908-3-59-S1.doc]

| 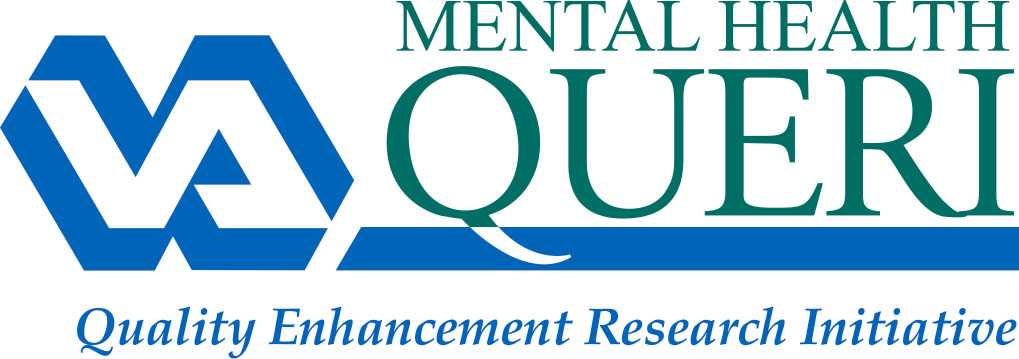 | 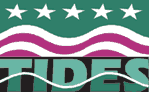 |
| --- | --- |

#### NATIONAL DISSEMINATION PLAN

#### PROGRESS REPORTING FORM

**Instructions:** As Leader of the Action Team for the TIDES National Dissemination Plan (NDP) goal listed below, please provide the information requested on the pages that follow. ***Please type your responses directly into the table (right-hand column), save and return by email to <NAME> at <Email address>. Please also forward copies of any important source documents prepared by yourself or other Action Team members in addressing this goal to <NAME> so they can be archived in Mental Health QUERI files.***

- Please be as thorough as possible in your responses, as this information will be used by the Mental Health QUERI Depression Subgroup and the ReTIDES Steering Committee to monitor progress in addressing NDP goals, ensure coordination of efforts across the different Action Teams, and identify any modifications to the NDP that may be needed.
- If you feel a question is not relevant to the NDP goal you are addressing, please type “not relevant” in the right-hand column of the table (please do not just leave the item blank).

**GUIDELINES AND** **QUALITY INDICATORS**

**Goal 1:** Partner with relevant VA offices/entities to update clinical practice guidelines for depression to reflect the evidence base for collaborative care.

| **QUESTION** | **ACTION TEAM LEADER RESPONSE** |
| --- | --- |
| VA Partners/Collaborators | |
| 1. **Which VA organizational leaders/stakeholders have been identified to date as key individuals with whom to engage or collaborate to accomplish the goal? What are their positions within the organization?** |  |
| 1. **What was the procedure for identifying the key VA organizational leaders/stakeholders in relation to this goal?** |  |
| 1. **Please describe the process for initiating contact and engaging with the identified leaders/stakeholders. What has worked? What didn’t work? What challenges (barriers) did you face, and how have they been addressed?** |  |
| Process | |
| 1. **Has the Action Team to address this goal been fully assembled? Please list all current members of the Action Team. Have you encountered any barriers in assembling the Action Team? If yes, how have the barriers been addressed?** |  |
| 1. **What is your action plan (in terms of process/procedure) for accomplishing the goal? *Please type your written action plan into the column to the right, or forward an electronic copy of the written plan to <NAME> at <Email Address> when you return this report.*** |  |
| 1. **Describe the process undertaken to date to accomplish the goal. *Please be sure to address each of the following questions (as applicable) in your response.***    - What **barriers or** **challenges** have been encountered? How have the barriers been addressed?    - What **facilitators** (in terms of people or new/existing infrastructure, policy initiatives, directives, etc) have positively influenced progress or success in meeting the goal?    - Has it been **necessary to** ***modify* the goal** during the process? If yes, why was this necessary? In what way was the original goal modified?    - Have any ***new goals*** for the Action Team to address emerged during the process? If yes, please list these new goals and explain why they are important.    - Have there been any **unintended consequences** in working to accomplish this goal? If yes, what were they? Were these unintended consequences positive or negative? |  |
| **Products** | |
| 1. **What planned product(s) (intermediate and/or final) will serve as indicator(s) of success in accomplishing the goal? Describe how these products will support national implementation.**     - Examples of products can include tangible products such as training materials, marketing materials/messages, and informatics tools, or intangible products such as new policies, modifications to existing policy, quality indicators, etc.    - An example of an intermediate product would be successful negotiation *at the VISN-level* of the establishment of a performance measure that rewards collaborative care for depression and/or serves as an indicator of fidelity in implementing the care model. An example of a final product would be *national adoption/implementation* of such a performance measure. | List intermediate (‘milestone’) products here:  List final products here: |
| 1. **Have any unplanned products emerged during the process of addressing the goal? What are those products? How will these products support national implementation?** |  |
| **Timeline** | |
| 1. **What is the timeline for accomplishing the goal?** |  |
| 1. **Based on progress to date, are you on schedule to accomplish the goal within the pre-specified timeframe? If no, why not?** |  |
| 1. **Were the timelines established initially to address this goal appropriate (knowing what you know now)? If no, what would be a more appropriate timeframe for accomplishing this goal?** |  |

# 
